# The Insecticide Imidacloprid Promotes Algal Growth in Absence of Zooplankton

**DOI:** 10.3390/jox15030090

**Published:** 2025-06-10

**Authors:** Verónica Laura Lozano, Florencia Soledad Alvarez Dalinger, Liliana Beatriz Moraña

**Affiliations:** 1 Consejo Nacional Investigaciones Científicas y Técnicas (CONICET), CCT Salta-Jujuy, Salta 4400, Argentina; floralvarezdalinger0@gmail.com; 2 Laboratorio de Calidad de Aguas (LaCA), Facultad de Ciencias Naturales, Universidad Nacional de Salta, Salta 4400, Argentina; lilymorana@gmail.com

**Keywords:** algae, imidacloprid, growth promotion, hormones

## Abstract

Imidacloprid, a systemic neonicotinoid insecticide, exerts its neurotoxic effects by binding to nicotinic acetylcholine receptors in the central nervous system. In this study, we examined the effects of commercial imidacloprid formulations on the growth of *Chlorella vulgaris* and other algal species, comparing these responses with those induced by plant hormones. Our results demonstrate that formulated imidacloprid stimulates *C. vulgaris* growth at concentrations as low as 7.82 μM, with a more pronounced effect than certain phytohormones. We observed similar growth-enhancing effects in other algal species exposed to imidacloprid. Notably, pure imidacloprid induced equivalent growth responses in *C. vulgaris*, confirming that the observed stimulation results from the active ingredient itself rather than formulation adjuvants. Given its insecticidal mode of action, potential worst-case aquatic contamination scenarios with imidacloprid may lead to significant increases in algal biomass through both direct (growth stimulation) and indirect (reduction of zooplankton grazing pressure) mechanisms.

## 1. Introduction

Imidacloprid, a systemic neonicotinoid insecticide, exerts its neurotoxic effects by binding to nicotinic acetylcholine receptors in the central nervous system of insects [1]. Beyond its insecticidal properties, growing evidence suggests that neonicotinoids may directly influence plant and algal physiology. While previous studies have documented increased crop yields following neonicotinoid application—even in the absence of insect pressure [2,3]—the underlying physiological mechanisms remain poorly characterized. Gonias et al. (2006) [3] proposed a reduction in stress temperature on cotton as a potential mechanism during imidacloprid treatments, although subsequent advancements in this area have been limited. More recently, Ford et al. (2010) [4] demonstrated that imidacloprid triggers salicylate-mediated defense responses in *Arabidopsis thaliana*. This finding is particularly intriguing given that salicylic acid, while generally inhibitory to vascular plant growth, has been shown to stimulate proliferation in algal species [5] and is now recognized as a novel phytohormone for microalgae like *Chlorella* sp. [6]. Further complexity arises from work by Lukaszewicz et al. (2019) [7], who reported that imidacloprid alters mitotic kinetics in the macrophyte *Bidens laevis* L., inducing significant increases in prophase cells, while reducing metaphase frequency.

The interaction between imidacloprid and algal systems remains underexplored, with most existing research focusing on microalgae for bioremediation purposes [8]. However, several studies have noted not just remarkable algal tolerance to imidacloprid, but actual growth stimulation at certain concentrations [9]. Freshwater ecosystems have been contaminated with imidacloprid at concentrations as high as 320 µg/L [10]. During routine ecotoxicological testing with *Chlorella vulgaris*, we made a counterintuitive observation: Significant growth enhancement occurred at high concentrations of commercial imidacloprid formulations. This prompted us to systematically test three hypotheses: (1) Imidacloprid induces growth stimulation comparable to known plant hormones in algae; (2) this growth-promoting effect is conserved across multiple algal species; and (3) the observed phenomenon is mediated by the active ingredient itself rather than formulation additives. Our findings may have important ecological implications, as imidacloprid contamination could potentially disrupt aquatic primary producer dynamics through direct growth modulation.

## 2. Materials and Methods

**Figure 1:**
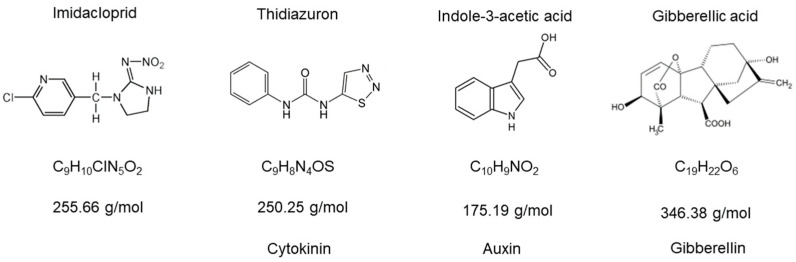
Molecular structures of imidacloprid and the plant hormones used in this work. The types of plant hormones selected highlight that we covered the most important modes of action.

Assays were carried out in 10 mL glass tubes using Bold medium. The culture chamber was maintained at 24 ± 1 °C and 12:12-h photoperiod; pH media was always ≈7. Imidacloprid was obtained in two distinct commercial formulations (Punto 3.5 of Gleba^®^ and GlacoXan^®^). Gibberellic acid, thidiazuron, and indoleacetic acid were procured from China as active principles (Figure 1). Statistical analyses were performed in R using linear mixed models with the packages nlme and emmeans. In general, as repeated measures were used in time, the models used in each assay followed this structure.

Model <- lme (Abs ~ Treatment x Day, random = ~1 | Tube)

Given that the variance was not equal across the different treatments, various models were constructed to account for this. The model with the lowest AIC and BIC values was selected; in all cases, the model with varIdent was finally chosen and used.

### 2.1. Assay 1: C. vulgaris Growth Under Different Imidacloprid Concentrations

Fifteen tubes with 8 mL of 1:10 Bold’s Basal Medium with *C. vulgaris* were utilized for treatments using the Gleba commercial formulation of imidacloprid Punto 3.5 (L.28161) purchased in Argentina. Treatments included control (0 mg/L) and 78.2 nM (0.02 mg/L), 0.782 μM (0.2 mg/L), 7.82 μM (2 mg/L), and 78.2 μM (20 mg/L) of imidacloprid. Treatments were conducted by triplicate and measured in spectrophotometer HACH DR5000 UV-Vis (Hach Company, Loveland, CO, USA) at 675 nm on days 0, 3, 4, 5, 7, and 10 post-treatments.

### 2.2. Assay 2: C. vulgaris Growth Under Imidacloprid and Plant Hormones Treatments

Eighteen tubes containing 8 mL of 1:10 Bold’s Basal Medium with *C. vulgaris* were employed for various treatments, including Control, Imidacloprid Gleba 78.2 μM, Imidacloprid GlacoXan (L.5074A purchased in Argentina) 78.2 μM, Thidiazuron 78.2 μM, Indolacetic acid 78.2 μM, and Gibberellic acid 78.2 μM. Triplicate treatments were evaluated at 675 nm on days 0, 1, 2, 3, 6, 7, 10, 13, 17, and 21 post-treatments. Additionally, one sample from each treatment was observed under 640× magnification using an inverted microscope after 24 h of sedimentation.

### 2.3. Assay 3: Growth Curves of Haematococcus pluvialis, Tetrademus sp., and Monoraphidium obtusa Under 7.82 μM of Imidacloprid Gleba Treatment

Isolated algae from Salta, Argentina, were cultivated in 8 mL of 1:10 Bold’s Basal Medium tubes. Treatments consisted of control and 78.2 μM of imidacloprid Gleba. Concentration was selected based on the highest observed growth effects on *C. vulgaris*, without signs of inhibition. Four replicates were performed for each treatment, and measurements were taken at 675 nm on days 0, 4, 6, 7, and 10.

### 2.4. Assay 4: Growth Response of C. vulgaris to Analytical Grade Imidacloprid

Due to the substantial cost difference between analytical-grade standards and commercial formulations, combined with funding limitations for scientific research in Argentina, we conducted a single confirmatory assay to establish whether the growth stimulation resulted from imidacloprid itself rather than formulation additives. To enhance treatment sensitivity, we used undiluted Bold’s Basal Medium (unlike previous experiments with 1:10 diluted medium). The assay employed imidacloprid analytical standard (Sigma-Aldrich, CAS 138261-41-3, PESTANAL^®^, St. Louis, MO, USA) at 78.2 μM, with seven biological replicates each for the treatment and control groups over a 10-day exposure period.

## 3. Results

### 3.1. C. vulgaris Growth Under Different Imidacloprid Concentrations (Assay 1)

**Figure 2:**
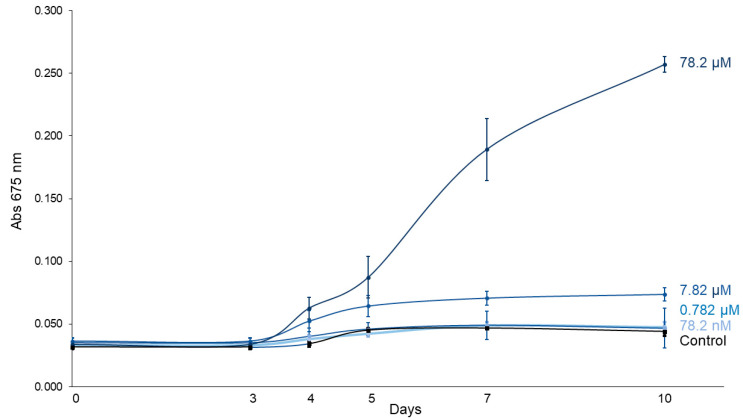
*C. vulgaris* growth curves under different concentrations of imidacloprid Gleba^®^ followed for 10 days.

Figure 2 displays the growth curves of *C. vulgaris* exposed to increasing concentrations of imidacloprid (Gleba^®^ formulation) over a 10-day period. A linear mixed-effects model showed that treatment (F_4,10_ = 83.56, *p* < 0.0001), time (F_5,50_ = 137.91, *p* < 0.0001) and their interaction (F_20,50_ = 46.19, *p* < 0.0001) significantly influenced *C. vulgaris* growth (Table 1), confirming differential growth responses depending on both concentration and exposure duration. Tukey post hoc contrasts revealed no significant differences in absorbance between treated and control groups during the early stages (days 0–3). However, the highest concentration (78.2 μM) significantly enhanced algal growth starting from day 4 (*p* = 0.0241), with increasingly stronger effects observed on days 5 (*p* = 0.0016), 7 (*p* < 0.0001), and 10 (*p* < 0.0001) (Table 2). Similarly, the 7.82 μM treatment also promoted growth from day 4 (*p* = 0.0180) onward. By day 10, the absorbance in the 78.2 μM treatment group was on average 5.84 times higher than in the control group, indicating a marked and concentration-dependent stimulatory effect of imidacloprid on *C. vulgaris* growth.

### 3.2. C. vulgaris Growth Under Imidacloprid Formulations and Plant Hormones Treatments (Assay 2)

**Figure 3:**
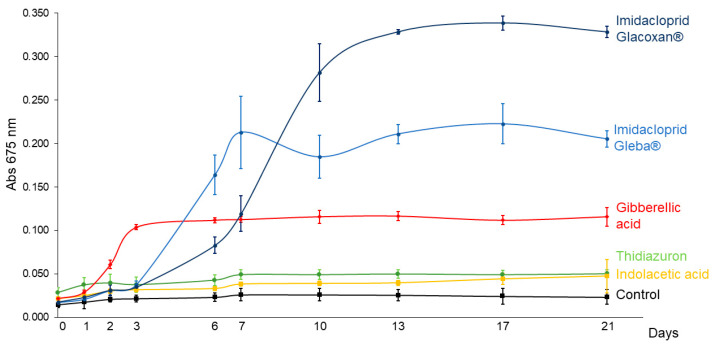
*C. vulgaris* growth curves under 78.2 μM of imidacloprid Glacoxan and Gleba commercial formulations, gibberellic acid, thidiazuron, and indolacetic acid. Cultures were followed up for 21 days. The naked eye showed more aggregation of cells under Gleba^®^ than under Glacoxan^®^ (Figure 4).

**Figure 4:**
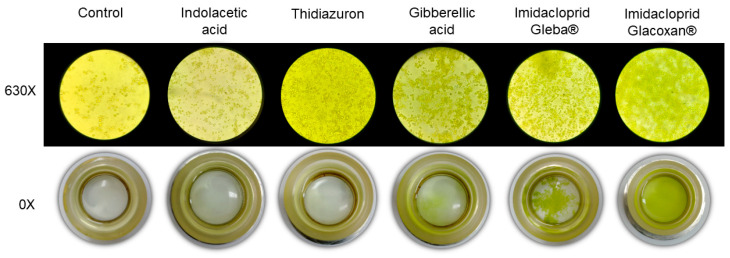
Inverted microscopy (630×) and sedimentation camaras (0×) at final time (21 days) for plant hormones and imidacloprid treatments at 78.2 μM.

To compare the growth-promoting effects of imidacloprid and plant hormones on *C. vulgaris*, we conducted a 21-day assay using a standardized concentration of 78.2 μM for all treatments. The tested compounds included two commercial formulations of imidacloprid (Gleba^®^ and Glacoxan^®^) and three plant hormones—gibberellic acid (GA), thidiazuron (TDZ), and indoleacetic acid (IAA)—to represent distinct hormonal modes of action. As shown in Figure 3 and Figure 4, the growth curves revealed differential responses among treatments. The linear mixed-effects model identified significant effects of treatment (F_5,17_ = 369.92, *p* < 0.0001), time (F_10,170_ = 176.09, *p* < 0.0001), and their interaction (F_50,170_ = 144.20, *p* < 0.0001), indicating treatment-specific dynamics over time (Table 3). Among the tested substances, *C. vulgaris* responded significantly only to gibberellic acid and the two imidacloprid formulations. GA induced an early growth promotion from day 3 (*p* = 0.0051), whereas Gleba^®^ and Glacoxan^®^ triggered significant responses later, from day 6 (*p* = 0.0004) and day 7 (*p* = 0.0015), respectively (Table 4). Notably, although Glacoxan^®^ acted one day later than Gleba^®^, it induced a stronger effect—by day 10, cultures treated with Glacoxan^®^ exhibited absorbance levels 1.6 times higher than those treated with Gleba^®^.

When comparing the imidacloprid formulations to gibberellic acid, both exhibited stronger long-term effects. By day 21, absorbance under Glacoxan^®^ and Gleba^®^ treatments was 2.84 and 1.77 times greater, respectively, than that observed with GA, suggesting that imidacloprid may mimic or amplify hormonal signaling pathways affecting algal growth.

These findings highlight the potent and delayed growth-stimulatory effects of imidacloprid compared to classical plant hormones and underscore the need to further investigate its mode of action in non-target photosynthetic organisms.

### 3.3. Growth Curves of Haematococcus pluvialis, Tetradesmus sp., and Monoraphidium obtusa Under 78.2 μM of Imidacloprid Gleba^®^ Treatment (Assay 3)

To assess whether the growth-promoting effect of imidacloprid observed in *Chlorella vulgaris* was consistent across other algal taxa, we exposed three freshwater green algae—*Haematococcus pluvialis*, *Tetradesmus* sp., and *Monoraphidium obtusa*—to 78.2 μM of the Gleba^®^ commercial imidacloprid formulation for 10 days.

**Figure 5:**
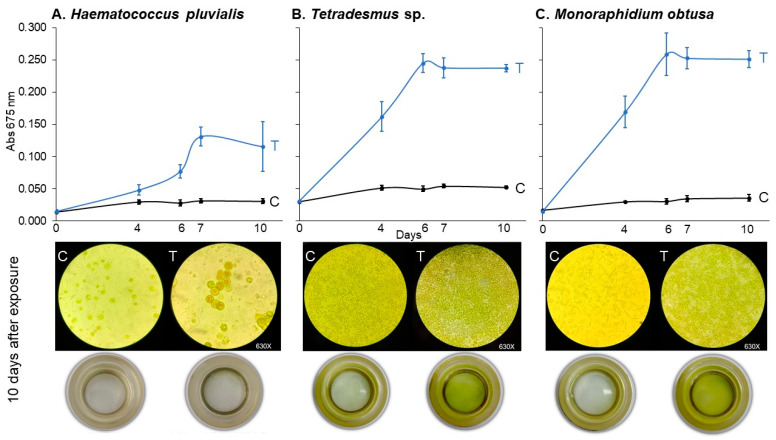
*Haematocccus pluvialis* (**A**), *Tetradesmus* sp. (**B**), and *Monoraphidium obtusa* (**C**) growth curves under 78.2 μM of imidacloprid Gleba^®^ commercial formulation for control (C) and treated (T) experimental units. Cultures were followed up for 10 days. Below, the inverted microscopy (630×) and sedimentation cameras (0×) photography are shown for the final time (10 days).

As shown in Figure 5, all three species exhibited significant growth stimulation, though with varying degrees of sensitivity and temporal response. The linear mixed-effects models confirmed significant effects of treatment (*p* < 0.001 for all species), time (*p* < 0.0001), and their interaction (*p* < 0.0001), indicating that absorbance trajectories differed across treatments over time (Table 5).

*Tetradesmus* sp. and *M. obtusa* were the most responsive species, showing statistically significant increases in growth from day 4 onward (*p* < 0.0001 for both; Table 6). In contrast, *H. pluvialis* displayed a delayed and weaker response, with significant differences emerging only from day 6 (*p* = 0.0037), and a comparatively smaller effect size throughout the assay period.

By day 10, all species treated with imidacloprid exhibited significantly greater absorbance values compared to controls (*p* ≤ 0.0003), confirming the broad but variable sensitivity of chlorophyte algae to the Gleba^®^ formulation.

These findings suggest that the growth-promoting effects of imidacloprid are not limited to *C. vulgaris* and may extend across phylogenetically diverse freshwater algae, albeit with species-specific dynamics in onset and magnitude of response.

### 3.4. Growth Response of C. vulgaris to Analytical-Grade Imidacloprid (Assay 4)

**Figure 6:**
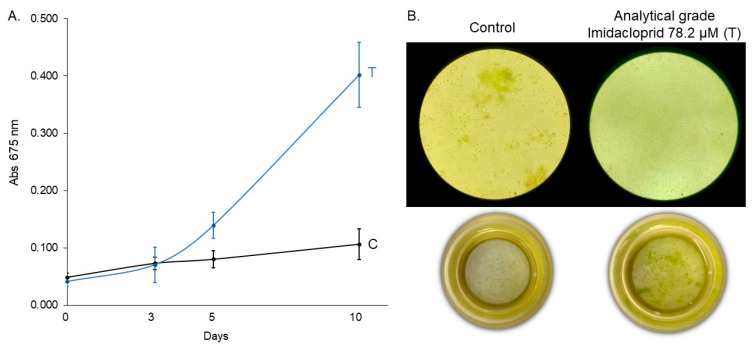
(**A**) Growth curve of *C. vulgaris* under control (C) and technical-grade imidacloprid treatment (T) over 10 days and (**B**) inverted microscopy (630×) and sedimentation chamber (0×) images at the final time point (10 days).

**Figure 7:**
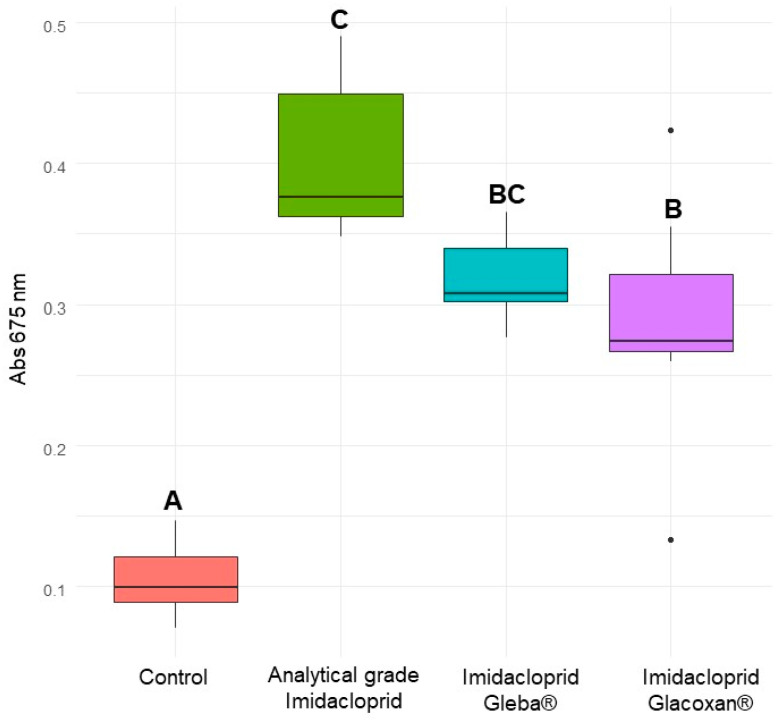
Absorbance of *C. vulgaris* at 675 nm after 10 days for control, analytical-grade imidacloprid (78.2 µM), imidacloprid Gleba^®^ (78.2 µM), and imidacloprid Glacoxan^®^ (78.2 µM). Different letters indicate significant differences in Tukey’s multiple comparison test (*p* < 0.05).

To verify that the observed growth stimulation in *C. vulgaris* was attributable specifically to imidacloprid and not to other ingredients or adjuvants present in the commercial formulation, we conducted an independent assay using 78.2 μM of technical-grade imidacloprid for 10 days. The linear mixed-effects model revealed a significant effect of treatment (F_1,42_ = 142.60, *p* < 0.0001), exposure time (F_3,42_ = 166.54, *p* < 0.0001), and their interaction (F_3,42_ = 97.55, *p* < 0.0001), indicating that both treatment and exposure duration influenced algal growth dynamics (Table 7). Post hoc Tukey comparisons showed no significant difference in absorbance between treated and control groups at days 0 and 3 (*p* = 0.6309 and *p* = 0.8752, respectively). However, from day 5 onwards, *C. vulgaris* exposed to technical-grade imidacloprid exhibited a significantly higher absorbance compared to the control (*p* = 0.0002 on day 5 and *p* < 0.0001 on day 10), suggesting a treatment-induced growth enhancement (Table 8, Figure 6). Finally, we compared the commercial formulations under identical experimental conditions. Analytical-grade imidacloprid exhibited the most significant growth-promoting effect, which was statistically indistinguishable from that of Gleba^®^ (Figure 7). These results confirm that the growth-promoting effect can be attributed to imidacloprid itself, independent of formulation components.

## 4. Discussion

Our results demonstrate that imidacloprid stimulates *C. vulgaris* growth more effectively than plant hormones at equivalent concentrations, an effect we observed across different algal families.

Some other studies have assessed the impact of imidacloprid on algal growth. For instance, Malev et al. (2012) [11] found no negative effect of imidacloprid within the range of 7.6 to 255.6 mg/L on *Desmodesmus subspicatus*, while its transformation product (6-chloronicotinic acid) had a detrimental effect. It is noteworthy that their study only measured up to 96 h, potentially limiting their ability to observe growth induction. The same limited range of time regarding algae growth is observed in Tisler et al. (2009) [12], where, also, *D. subspicatus* was assayed only for 72 h.

Deng et al. (2022) [9] similarly reported that imidacloprid promoted *C. vulgaris* growth between 10 and 50 mg/L. In our study, we observed a similar growth-promoting effect at concentrations of 2 and 20 mg/L (7.82 and 78.2 μM, respectively). However, Deng. et al. suggested that this effect might be attributed to the utilization of imidacloprid as a carbon source by *C. vulgaris*. While we cannot discard this possibility, the use of alternatives carbon sources in algae is not a common characteristic and was not reported before for *C. vulgaris*. For instance, we have assayed the effect of imidacloprid formulations in Erlenmeyer’s with continues agitation for avoiding CO_2_ limitation and the promotion of growth was constated. The removal of imidacloprid using algae has been documented in multiple studies [8,13,14,15,16] highlighting the presence of incorporation mechanisms and molecular pathways involving imidacloprid in algae, unfortunately without biotransformation products analysis. The molecular resemblance to cytokinins suggests a potential hormonal function, as cytokinin pathways have been reported in algae [17,18]. Considering that imidacloprid is a neonicotinoid, it is important to study a potential hormonal pathway linked to nicotine, particularly, the jasmonic acid pathway. Further research is necessary to elucidate the underlying mechanism.

From an ecotoxicological perspective, imidacloprid is well known for its pronounced toxicity to primary consumers such as zooplankton. Indirect stimulation of algal blooms through the suppression of grazers has been observed under low concentrations of imidacloprid in mesocosm studies [19]. Thus, trophic cascade effects may represent the primary ecological mechanism through which imidacloprid alters community dynamics in freshwater systems [20]. Nevertheless, our findings suggest that direct stimulation of algal growth by imidacloprid could also play a role—particularly in high-contamination scenarios—adding an additional layer of complexity to its ecological impacts.

## 5. Conclusions

High concentrations of imidacloprid promote algal growth, potentially heightening aquatic toxicological risks under worst-case scenarios. As this effect exceeds that of certain plant hormones, further research is needed to uncover the underlying molecular mechanisms.

## Figures and Tables

**Table 1:** Summary of the lineal mixed model obtained for the absorbance (675 nm) in *C. vulgaris* growth curves under different concentrations of imidacloprid Gleba^®^. Statistically significant *p*-values (<0.05) are shown in bold.

	numDF	denDF	F-Value	*p*-Value
**(Intercept)**	1	50	1882.23	**<0.0001**
**Treatment**	4	10	83.56	**<0.0001**
**Day**	5	50	137.91	**<0.0001**
**Treatment–Day**	20	50	46.19	**<0.0001**

**Table 2:** Tukey contrasts between different concentrations of imidacloprid and the control group. Statistically significant *p*-values (<0.05) are shown in bold.

Day	78.2 nM	0.782 µM	7.82 μM	78.2 μM
**0**	0.9960	1.0000	0.9955	1.0000
**3**	0.9960	0.9363	0.8387	0.9987
**4**	0.8595	0.7276	**0.0180**	**0.0241**
**5**	0.9493	0.9995	**0.0115**	**0.0016**
**7**	0.9681	0.9871	**0.0026**	**<0.0001**
**10**	0.8595	0.9790	**0.0005**	**<0.0001**

**Table 3:** Summary of the lineal mixed model obtained for the absorbance (675 nm) in *C. vulgaris* growth curves under 78.2 μM concentrations of imidacloprid in two commercial formulations, gibberellic acid, thidiazuron, and indolacetic acid. Statistically significant *p*-values (<0.05) are shown in bold.

	numDF	denDF	F-Value	*p*-Value
**(Intercept)**	1	170	3489.65	**<0.0001**
**Treatment**	5	17	369.92	**<0.0001**
**Day**	10	170	176.09	**<0.0001**
**Treatment–Day**	50	170	144.20	**<0.0001**

**Table 4:** Tukey contrasts between treatments and the control group. Statistically significant *p*-values (<0.05) are shown in bold.

Day	Indolacetic Acid	Thidiazuron	Gibberellic Acid	Imidacloprid Gleba^®^	Imidacloprid Glacoxan^®^
**0**	0.9990	0.9734	0.9989	1.0000	1.0000
**1**	0.9987	0.8953	0.9898	1.0000	0.9996
**2**	0.9960	0.9151	0.3336	0.9985	0.9928
**3**	0.9940	0.9536	**0.0051**	0.9907	0.9790
**6**	0.9944	0.8985	**0.0026**	**0.0004**	0.0551
**7**	0.9855	0.8181	**0.0031**	**<0.0001**	**0.0015**
**10**	0.9811	0.8181	**0.0022**	**0.0001**	**<0.0001**
**13**	0.9734	0.7894	**0.0020**	**<0.0001**	**<0.0001**
**15**	0.9108	0.7639	**0.0022**	**<0.0001**	**<0.0001**
**17**	0.8937	0.7733	**0.0028**	**<0.0001**	**<0.0001**
**21**	0.7939	0.7150	**0.0017**	**<0.0001**	**<0.0001**

**Table 5:** Summary of the lineal mixed models obtained for the absorbance (675 nm) of *Haematococcus pluvialis*, *Tetradesmus* sp., and *Monoraphidium obtusa* under 78.2 μM concentrations of imidacloprid Gleba^®^. Statistically significant *p*-values (<0.05) are shown in bold.

		numDF	denDF	F-Value	*p*-Value
	(Intercept)	1	20	447.30	**<0.0001**
***H. pluvialis***	Treatment	1	5	83.99	**0.0003**
	Day	4	20	53.17	**<0.0001**
	Treatment–Day	4	20	14.51	**<0.0001**
	(Intercept)	1	24	2860.91	**<0.0001**
***Tetradesmus *** **sp.**	Treatment	1	6	1406.98	**<0.0001**
	Day	4	24	187.59	**<0.0001**
	Treatment–Day	4	24	138.47	**<0.0001**
	(Intercept)	1	24	930.68	**<0.0001**
	Treatment	1	6	1013.19	**0.0003**
***M. obtusa***	Day	4	24	61.09	**<0.0001**
	Treatment–Day	4	24	88.54	**<0.0001**

**Table 6:** Tukey contrasts between treatments and the control group for each specie. Statistically significant *p*-values (<0.05) are shown in bold.

Day	*Haematococcus pluvialis*	*Tetrademus* sp.	*Monoraphidium obtusa*
**0**	0.9009	0.8678	0.8910
**4**	0.1729	**<0.0001**	**<0.0001**
**6**	**0.0037**	**<0.0001**	**<0.0001**
**7**	**0.0001**	**<0.0001**	**<0.0001**
**10**	**0.0003**	**<0.0001**	**<0.0001**

**Table 7:** Summary of the lineal mixed model obtained for the absorbance (675 nm) of *C. vulgaris* grown under 78.2 μM of technical-grade imidacloprid. Statistically significant *p*-values (<0.05) are shown in bold.

	numDF	denDF	F-Value	*p*-Value
**(Intercept)**	1	42	1106.68	**<0.0001**
**Treatment**	1	42	142.60	**<0.0001**
**Day**	3	42	166.54	**<0.0001**
**Treatment–Day**	3	42	97.55	**<0.0001**

**Table 8:** Tukey contrasts between *C. vulgaris* grown under 7.82 μM of technical-grade imidacloprid and the control group by time. Statistically significant *p*-values (<0.05) are shown in bold.

Day	78.2 μM
**0**	0.6309
**3**	0.8752
**5**	**0.0002**
**10**	**<0.0001**

## Data Availability

The original contributions presented in this study are included in the article. Further inquiries can be directed to the corresponding author(s).

## References

[1] Sheets L.P. (2010). Imidacloprid: A neonicotinoid insecticide. Hayes’ Handbook of Pesticide Toxicology.

[2] Senn R., Hofer D., Thieme T., Zang L. (2004). Method for Improving Plant Growth. U.S. Patent.

[3] Gonias E.D., Oosterhuis D.M., Bibi A.C. How the insecticide Trimax TM improves the growth and yield of cotton. Proceedings of the Beltwide Cotton Conference.

[4] Ford K.A., Casida J.E., Chandran D., Gulevich A.G., Okrent R.A., Durkin K.A., Bunnelle E.M., Wildermuth M.C. (2010). Neonicotinoid insecticides induce salicylate-associated plant defense responses. Proc. Natl. Acad. Sci. USA.

[5] Awad N., Vega-Estévez S., Griffiths G. (2020). Salicylic acid and aspirin stimulate growth of Chlamydomonas and inhibit lipoxygenase and chloroplast desaturase pathways. Plant Physiol. Biochem..

[6] Fu L., Li Q., Chen C., Zhang Y., Liu Y., Xu L., Zhou Y., Li C., Zhou D., Rittmann B.E. (2021). Benzoic and salicylic acid are the signaling molecules of Chlorella cells for improving cell growth. Chemosphere.

[7] Lukaszewicz G., Iturburu F.G., Garanzini D.S., Menone M.L., Pflugmacher S. (2019). Imidacloprid modifies the mitotic kinetics and causes both aneugenic and clastogenic effects in the macrophyte *Bidens laevis* L.. Heliyon.

[8] Cheng Y., Wang H., Deng Z., Wang J., Liu Z., Chen Y., Ma Y., Li B., Yang L., Zhang Z. (2022). Efficient removal of Imidacloprid and nutrients by algae-bacteria biofilm reactor (ABBR) in municipal wastewater: Performance, mechanisms and the importance of illumination. Chemosphere.

[9] Deng Z., Zhu J., Yang L., Zhang Z., Li B., Xia L., Wu L. (2022). Microalgae fuel cells enhanced biodegradation of imidacloprid by *Chlorella* sp.. Biochem. Eng. J..

[10] Van Dijk T.C., Van Staalduinen M.A., Van der Sluijs J.P. (2013). Macro-invertebrate decline in surface water polluted with imidacloprid. PLoS ONE.

[11] Malev O., Klobučar R.S., Fabbretti E., Trebše P. (2012). Comparative toxicity of imidacloprid and its transformation product 6-chloronicotinic acid to non-target aquatic organisms: Microalgae Desmodesmus subspicatus and amphipod Gammarus fossarum. Pestic. Biochem. Physiol..

[12] Tišler T., Jemec A., Mozetič B., Trebše P. (2009). Hazard identification of imidacloprid to aquatic environment. Chemosphere.

[13] Encarnação T., Santos D., Ferreira S., Valente A.J., Pereira J.C., Campos M.G., Burrows H.D., Pais A.A. (2021). Removal of imidacloprid from water by microalgae Nannochloropsis sp. and its determination by a validated RP-HPLC method. Bull. Environ. Contam. Toxicol..

[14] Cheng Y., Liu Z., Wang J., Wu L. (2022). Study on Efficient Removal of Imidacloprid and Nutrients from Sewage by *Chlorella* sp.. Environ. Sci. Technol..

[15] Li D., Li J., Yeerhazi B., Cheng Y. (2023). Efficient removal of imidacloprid from sewage by Scenedesmus sp. TXH and the effects of environmental factors on its removal. J. Chem. Technol. Biotechnol..

[16] Reyad A.G., Abbassy M.A., Marei G.I.K., Rabea E.I., Badawy M.E. (2023). Removal of fenamiphos, imidacloprid, and oxamyl pesticides from water by microalgal Nannochloropsis oculata biomass and their determination by validated HPLC method. J. Environ. Sci. Health Part B.

[17] Stirk W.A., Ördög V., Van Staden J., Jäger K. (2002). Cytokinin-and auxin-like activity in Cyanophyta and microalgae. J. Appl. Phycol..

[18] Pinto S., Tajeshwar N., Gordon K., Borrero P., Novák O., Strnad M., Foellmer M., Heyl A. (2023). Cytokinin Response of the Streptophyte Alga Coleochaete scutata provides a clue to the evolution of cytokinin signaling. Front. Plant Physiol..

[19] Yao K.S., Li D., Lei H.J., Van den Brink P.J., Ying G.G. (2021). Imidacloprid treatments induces cyanobacteria blooms in freshwater communities under sub-tropical conditions. Aquat. Toxicol..

[20] Sumon K.A., Ritika A.K., Peeters E.T., Rashid H., Bosma R.H., Rahman M.S., Fatema M.K., Van den Brink P.J. (2018). Effects of imidacloprid on the ecology of sub-tropical freshwater microcosms. Environ. Pollut..

